# Presynaptic Changes in Mouse Rod Photoreceptors During Early Retinitis Pigmentosa

**DOI:** 10.1167/iovs.66.15.4

**Published:** 2025-12-01

**Authors:** Elias Roihuvuo, Lee Sturgis, Ahmed B. Montaser, Marcin Tabaka, Elliot H. Choi, Deepa Mathew, Anthi-Styliani Makiou, Kristiina M. Huttunen, Krzysztof Palczewski, Frans Vinberg, Henri Leinonen

**Affiliations:** 1 School of Pharmacy, Faculty of Health Sciences, University of Eastern Finland, Kuopio, Finland; 2 International Centre for Translational Eye Research, Warsaw, Poland; 3 Institute of Physical Chemistry, Polish Academy of Sciences, Warsaw, Poland; 4 Department of Ophthalmology, Gavin Herbert Eye Institute, University of California, Irvine, California, United States; 5 John A. Moran Eye Center, University of Utah, Salt Lake City, Utah, United States; 6 Department of Physiology and Biophysics, University of California – Irvine, Irvine, California, United States; 7 Department of Chemistry, University of California – Irvine, Irvine, California, United States; 8 Department of Molecular Biology and Biochemistry, University of California – Irvine, Irvine, California, United States

**Keywords:** rods, plasticity, synaptic transmission, retinitis pigmentosa (RP), rod bipolar cells

## Abstract

**Purpose:**

Homeostatic plasticity is crucial for maintaining stable neural activity by adjusting strength and intrinsic properties of synapses. This mechanism is vital for normal nervous system function and plays a role in various neurological conditions, including retinal degenerations. Sensitive night vision has been shown in P23H/*Gnat2^−^^/^^−^* retinitis pigmentosa (RP) mice, which lack cone phototransduction and rely solely on rods, even after losing more than half of their rod photoreceptors. While homeostatic plasticity has been proposed as a potential explanation, the underlying molecular mechanisms remain unclear. The aim of this study was to investigate the molecular basis of this phenomenon.

**Methods:**

Single cell RNA-sequencing (scRNA-seq) and bulk retina proteomics were used to investigate the transcriptomic and proteomic changes of the degenerating retinas in 1-month-old P23H/*Gnat2^−^^/^^−^* RP and *Gnat2^−^^/^^−^* control mice. Immunohistochemistry was used to analyze the expression of synaptic SNARE complex and vesicle proteins, SNAP25 and SYT1, in the outer plexiform layer, the site of rod axon terminals.

**Results:**

This study shows a significant upregulation of genes encoding synaptic SNARE complex and vesicle proteins (*Snap25*, *Stxbp1*, and *Syt1*) in P23H mouse rods. Bulk retina proteomics analysis shows trends toward upregulation of the corresponding proteins as well as upregulation of many matrix-associated and trans-synaptic-complex proteins. Immunohistochemistry shows persistent SYT1 and SNAP25 expression in the outer plexiform layer of P23H/*Gnat2^−^*^/^*^−^* mice despite significant rod death.

**Conclusions:**

Rod degeneration induces molecular changes in the P23H/*Gnat2^−^*^/^*^−^* mouse rods that suggest synaptic plasticity and strengthening of rod-rod bipolar cell synaptic transmission in early RP.

Homeostatic plasticity is a regulatory mechanism in the nervous system that maintains stable neural activity despite changes in input or network conditions.^1^^,^^2^ This feedback system ensures that neurons do not become overly excitable nor too inactive. Homeostatic plasticity can be manifested through multiple mechanisms such as the following: (1) synaptic scaling adjusts the strength of synapses proportionally to maintain overall activity. This scaling can originate from a presynaptic mechanism in which neurotransmitter release is adjusted to maintain postsynaptic activity to compensate for loss of presynaptic cells or postsynaptic receptors.^3^ The scaling can also have postsynaptic origins, where the neuron's activity can be stabilized by increasing or decreasing the strength of its excitatory synapses.^4^ (2) Intrinsic plasticity alters a neuron’s excitability by modifying receptor or ion-channel expression, or other properties. (3) Structural plasticity can be achieved by changing the number of synaptic connections to balance the activity. Homeostatic plasticity is vital for normal functioning of the nervous system, but it also plays an important role in multiple pathological states.^5^^–^^7^

Homeostatic plasticity can be triggered in several types of sensory impairments, including retinal degeneration and hearing loss^8^^–^^13^; as well as in association with neurodegenerative diseases, such as myasthenia gravis,^14^^,^^15^ amyotrophic lateral sclerosis (ALS),^16^ and Alzheimer's and Parkinson's diseases.^17^ It is postulated that homeostatic plasticity can support physiological functions during disease states in the neuromuscular junction,^8^^,^^17^ cochlea,^18^ and retina.^8^ In ALS, variations in disease onset and progression may be partly dependent on how strongly presynaptic homeostatic plasticity is expressed.^19^

Previous work from the mouse retina has demonstrated compensatory signaling potentiation between sensory rod photoreceptors and their postsynaptic partners, rod bipolar cells (RBCs), during retinitis pigmentosa (RP) caused by the P23H rhodopsin mutation.^20^ This compensation is manifested as high pattern contrast sensitivity at dim light even after 50% loss of the rod population. Several other studies have later found similar compensatory phenomena in different RP mouse models,^21^^,^^22^ as well as in other retinal dystrophy models.^23^^,^^24^ In degenerating retinas with nonfunctional rods, RBCs can establish a connection with the remaining cones, thereby improving retinas’ light responsiveness.^25^ Taken together, there is considerable therapeutic potential in targeting homeostatic plasticity in retinal degeneration. However, more mechanistic research in different disease paradigms is required for rational design of therapeutic interventions.

## Methods

### Animal Model

This study utilized the P23H/*Gnat2^−^^/^^−^* double knock-in/knock-out mouse model.^20^ The P23H mouse carries an autosomal-dominant mutation in rhodopsin, which serves as a well-established model of RP that closely mimics key features of the human disease. Knock-in techniques were used to introduce the P23H-opsin mutation.^26^ Given the study's focus on the rod pathway and scotopic vision, cone-transducin knockout (*Gnat2^−^^/^^−^*) mice were used as the background strain. In *Gnat2^−^^/^^−^* mice, cone phototransduction is abolished whereas retinal anatomy remains preserved, with no evidence of retinal remodeling observed for up to at least 9 months of age.^27^ The experimental P23H-heterozygote/*Gnat2^−^^/^^−^* and littermate *Gnat2^−^^/^^−^* control mice used in this study were generated using P23H-heterozygote/*Gnat2^−^^/^^−^* and wild-type/*Gnat2^−^^/^^−^* breeding pairs.^20^ All animal procedures were conducted in accordance with the Council of Europe (Directive 86/609) and Finnish guidelines, and were approved by the Animal Experiment Board of Finland. Experiments adhered to the ARVO Statement for the Use of Animals in Ophthalmic and Vision Research.

### Analysis of Single Cell RNA-Sequencing Data

Single-cell RNA sequencing (scRNA-seq) was performed on retinas from four *Gnat2^−^^/^^−^* and four P23H/*Gnat2^−^^/^^−^* mice at 1 month of age. Sexes were balanced within each group, with two male and two female mice included. The mice were euthanized by cervical dislocation, and one retina from each mouse was dissociated into single-cell suspensions using papain-mediated enzymatic digestion, and sequencing was conducted using the Genomics 10 × platform as previously described.^28^

The pre-processing steps—including the generation and demultiplexing of FASTQ files from raw sequencing reads (bclfastq, version 2.20), aligning to the University of California Santa Cruz (UCSC) mm10 transcriptome, and the generation of raw count matrices were performed using Cell Ranger (version 6.0.1), with default settings. Expression matrices were combined using Cumulus version 1.5.0,^29^ and cell doublets were identified and removed with Scrublet version 0.2.1.^30^ All computational pipelines were executed on the Terra Cloud Platform (https://app.terra.bio/). Downstream analysis was carried out in Seurat version 3.2.2 following the standard workflow, retaining cells expressing more than 200 genes, resulting in 5805 cells from *Gnat2^−^^/^^−^* mice and 7266 cells from P23H/*Gnat2^−^^/^^−^* mice.^31^ Principal component analysis (PCA) was performed on the top 2000 most-variable genes identified using the FindVariableGenes function in Seurat. Batch effects across experimental groups were mitigated using the Harmony package version 0.1.0 to remove non-cell-type-specific factors that may impact clustering.^32^ The number of top principal components (PCs) was assessed by the elbow method, keeping 25 PCs for clustering and data visualization. Cells were clustered using a shared nearest-neighbor modularity optimization-based algorithm (FindClusters in Seurat). Cluster-specific genes were identified using FindAllMarkers in Seurat, using the MAST test with the number of unique molecular identifiers (UMIs) detected as a latent variable.^33^ Differentially expressed genes (DEGs) between experimental groups within cell clusters were identified using the same test. Established retinal cell-type-specific markers were used to annotate cell clusters (Supplementary File S1).^34^

### Bulk Retina Proteomics

Full retinal extracts were prepared from 1-month-old *Gnat2^−^^/^^−^* mice (*n* = 5) and P23H/*Gnat2^−^^/^^−^* mice (*n* = 10) using both retinas from each mouse. Following direct cervical dislocation of the mice, fresh mouse retinas were dissected in ice-cold PBS, snap-frozen, and stored at −80°C, as previously described.^20^ Retinas were homogenized in 100 µL of protein-extraction buffer (ab193970; Abcam) using 3 cycles of probe sonication (10 seconds per cycle, Soniprep 150), with 10-second intervals on ice between cycles. The homogenates were centrifuged at 18,000*g* for 20 minutes at 4°C, and the resulting supernatants, containing solubilized proteins, were collected in low-protein-binding Eppendorf tubes (Thermo Fisher Scientific). Total protein concentration was determined using the bicinchoninic acid (BCA) protein assay (Thermo Fisher Scientific). The extracted and solubilized retinal proteins were processed following an in-solution digestion protocol, as previously described.^35^ Briefly, 50 µg of protein was mixed with a denaturing solution containing 7 M guanidine hydrochloride, 0.5 M Tris-HCl, and 10 mM EDTA-Na. Proteins were then reduced with dithiothreitol (1:50, w/w) and S-carboxymethylated with iodoacetamide (1:20, w/w; Sigma-Aldrich, St. Louis, MO, USA). The alkylated proteins were precipitated using a methanol/chloroform/water mixture (4/1/3) and centrifuged at 18,000*g* for 5 minutes at 4°C. The resulting pellet was resuspended in 6 M urea and mixed for 10 minutes at room temperature. The samples were then diluted with 0.1 M Tris-HCl to a final urea concentration of 1.2 M. Samples were fully dissolved using intermittent sonication (Branson 3510, Danbury, CT, USA). Proteolytic digestion was performed in two stages: initially with LysC (1:100, w/w; Sigma-Aldrich, St. Louis, MO, USA) in the presence of 0.05% ProteaseMax (Promega Biotech AB, Nacka, Sweden) for 3 hours at room temperature, followed by digestion with TPCK-treated trypsin (1:100, w/w; Promega Biotech AB, Nacka, Sweden) for 18 hours at 37°C. The reaction was quenched by adding 40 µL of 5% formic acid (FA) to a final protein concentration of 0.5 µg/µL, and samples were centrifuged at 18,000*g* for 5 minutes at 4°C. The supernatant was then desalted using an Oasis HLB Cartridge (Waters) according to the manufacturer's protocol, and samples were concentrated in vacuo, using a SpeedVac vacuum concentrator (Thermo Fisher Scientific, Waltham, MA, USA). Samples were resuspended in 100 µL of 2% acetonitrile (ACN) acidified with 0.1% FA in MilliQ water by mixing at 800 rpm for 15 minutes. Samples were briefly centrifuged at 18,000*g* for 2 minutes and supernatants were transferred to high-performance liquid chromatography (HPLC) vials.

Liquid chromatography–mass spectrometry (LC-MS) was conducted as previously described.^36^ Proteomics analysis was performed using a Vanquish Flex UPLC (Thermo Scientific) coupled to a high-resolution Orbitrap Q Exactive Classic mass spectrometer (Thermo Scientific) in positive ion mode. Mobile phase A consisted of 0.1% FA in water, and mobile phase B was 0.1% FA in ACN. Peptides were loaded onto an Agilent AdvanceBio Peptide Map column (2.1 mm × 250 mm, 2.7 µm) and separated at 0.3 mL/min over an 80-minute gradient (2–45% B; total run time 90 minutes). MS detection included full MS–single ion monitoring (SIM; 35k resolution, AGC target 3e6, max injection time 60 ms, and 385–1015 m/z) and data-independent acquisition (DIA; 17.5k resolution, AGC target 2e6, max injection time 60 ms, loop count 25, and 24 m/z isolation window). The raw data were processed using DIA-NN (Data-Independent Acquisition by Neural Networks) software (version 1.8) in library-free DIA mode, with default parameters.^37^ Peptide tandem mass spectrometry (MS/MS) spectra and retention times were predicted based on the UniProt-mouse reference proteome (UP000000589_10090 and UP000000589_10090_additional FASTA, June 2021; 63,534 proteins). Cysteine residues were set as static modifications, whereas methionine oxidation and N-terminal acetylation were set as variable modifications, with a maximum of two per peptide. The predicted MS library was used for data searching, applying 1% false discovery rate (FDR) thresholds at the precursor and protein group levels, and requiring at least one proteotypic peptide (7–30 amino acids). Label-free quantified (LFQ) and MaxLFQ-normalized protein intensities were used for data evaluation and downstream analysis.^38^

The proteins of interest were selected primarily based on their established or possible roles in presynaptic scaling of rod photoreceptors.^39^ Additionally, subsets of proteins associated with postsynaptic scaling of RBCs or the strengthening of matrix-associated transsynaptic complexes were included, based on scRNA-seq data and their potential involvement in postsynaptic scaling or trans-synaptic integration.

### Immunohistochemistry, Microscopy, and Image Analysis

Mice were euthanized by carbon dioxide asphyxiation followed by cervical dislocation. Eyes from 1-month-old *Gnat2^−^^/^^−^* (*n* = 5, 5–6 retinas used) and P23H/*Gnat2^−^^/^^−^* (*n* = 7, 7–8 retinas used) mice were enucleated and immediately fixed in ice-cold 4% paraformaldehyde (PFA). Corneas were removed, and the eyes were kept in 4% PFA for 20 minutes on wet ice. Then, the eyes were rinsed in 1 × phosphate buffered saline (PBS) and lenses were removed. Eyecups were cryoprotected in a sucrose/PBS gradient (10%, 20%, and 30%) each time until the eyecups sank; after which they were placed in a 1:2 solution of 20% sucrose/PBS and optimal cutting temperature (OCT) compound (Sakura Finetech) mix overnight at 4°C. Eyes were embedded in 20% sucrose/PBS and OCT mix and cryosectioned at 10 µm. Slides were rinsed 3 × 10 minutes in PBS to remove OCT. A blocking buffer made of 0.1% Triton X-100 (Fisher Scientific) and 10 % normal goat serum in PBS was used to block nonspecific binding for 1 hour at room temperature. Samples were incubated with primary antibody overnight in a humidified dark chamber on an orbital shaker at 4°C. The following day, the slides were rinsed 3 × 10 minutes in PBS, and then incubated with secondary antibody for 2 hours in darkness on an orbital shaker at room temperature. Slides were then rinsed 3 × 10 minutes in PBS, and mounted with DAPI Fluoromount-G (SouthernBiotech, Cat. No.: 0100-20) and sealed under cover slips. Primary antibodies used were mouse monoclonal anti-synaptotagmin-1 antibody (Synaptic Systems Antibodies, Cat. No.: 105011, dilution 1:500), rabbit recombinant monoclonal anti-SNAP25 antibody (Abcam, Cat. No.: ab109105, dilution 1:250), and rabbit recombinant monoclonal anti-Munc18-1 antibody (Abcam, Cat. No.: ab109023, dilution 1:100). Secondary antibodies used were goat anti-mouse secondary antibody, Alexa Fluor 488 (Invitrogen, Cat. No.: A11029, dilution 1:2000), or goat anti-rabbit secondary antibody, Alexa Fluor 647 (Invitrogen, Cat. No.: A-21245, dilution 1:1000).

A Leica THUNDER Imager 3D Tissue-slide scanner (Wetzlar, Germany) with a 20 × objective was used to conduct fluorescence light microscopy. LAS X software (version 3.7.5.24914) was used to adjust image brightness and contrast. Images were analyzed using ImageJ software (version 1.54*g*) so that, based on negative controls, pixels with a value of 0 were excluded from the mean pixel brightness analysis. Clearly overexposed areas due to artifacts, such as those due to breaking or folding of the section, were also excluded.

### Statistical Analysis

Perseus software (version 1.6.5.0) was used to preprocess the global proteomics data.^40^ LFQ values were log2 transformed, and proteins were retained only if at least three valid values (>0) occurred in both experimental groups. Missing values were imputed using Perseus's built-in imputation function, which sampled from a normal distribution. Differential expression analysis was performed using the Linear Models for Microarray Analysis (limma) package and corrected for multiple comparisons by applying FDR and setting the significance level at 0.05.

For visualization and additional statistical testing, raw LFQ intensities (approximately relative expression level) in the raw global proteomics data (before processing in Perseus) were normalized to the mean LFQ value of the control group and imported into GraphPad Prism (version 10.4.1). A two-tailed Mann-Whitney *U* test, corrected for multiple comparisons using the two-stage step-up method of Benjamini, Krieger, and Yekutieli (FDR = 0.05) was used to perform statistical comparisons. Differences in the mean pixel brightness were tested using an unpaired, two-tailed Welch's *t*-test.

### Data Availability

The scRNA-seq data generated in this study have been deposited in the NCBI GEO database under accession code GSE291972. The raw and processed scRNA-seq data are provided in the Supplementary Information and Supplementary Data files. The MS proteomics data have been deposited to the ProteomeXchange Consortium^41^ via the skyline and Panorama public repository with the dataset identifier PXD060180.

## Results

### Single-Cell RNA Sequencing Suggests Presynaptic Rather Than Postsynaptic Scaling in P23H/Gnat2*^−^*^/^*^−^* Mouse Rod-RBC Synapses

In our earlier work, we used a cone transducin knockout (P23H/*Gnat2^−^^/^^−^*) RP model to isolate rod-driven pathology and demonstrated broad transcriptomic changes via bulk retina RNA-seq, implicating pathways such as synaptic organization, axonogenesis, and cell adhesion.^20^ However, bulk RNA-seq can mask cell-type-specific alterations critical for understanding early disease mechanisms. Here, we overcome this limitation by applying scRNA-seq to precisely resolve transcriptomic changes within individual retinal cell populations, specifically focusing on the rods and RBCs in 1-month-old P23H/*Gnat2^−^^/^^−^* mice versus *Gnat2^−^^/^^−^* controls. This approach provides a high-resolution map of cell-type-specific molecular remodeling in early RP, offering insights unattainable through prior bulk analyses.

**Figure 1.**
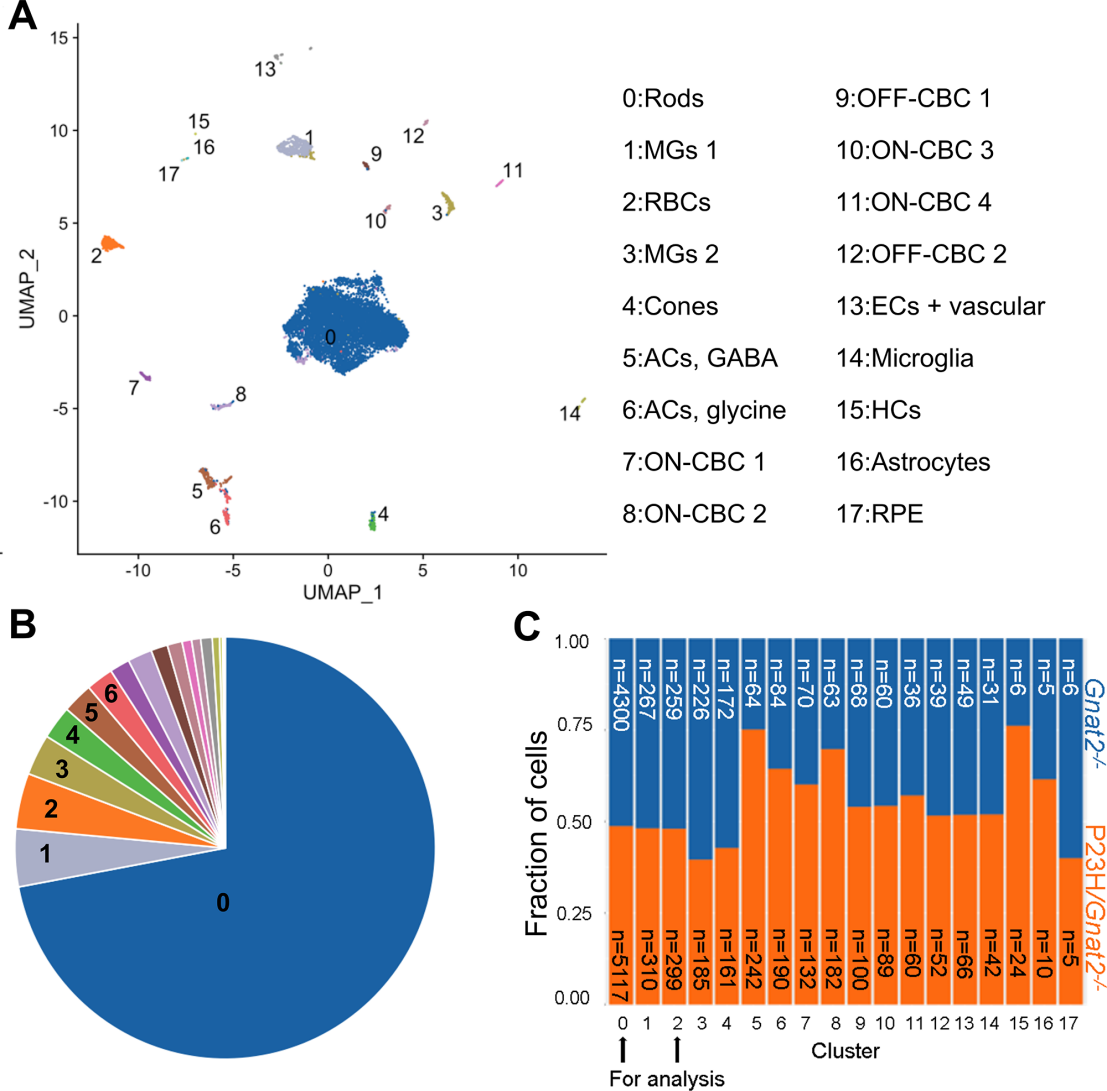
**Cell-cluster characteristics from single cell RNA-sequencing (scRNA-seq).** Single-cell suspensions for scRNA-seq were prepared from the retinas of control mice (*Gnat2^−^^/^^−^*, *n* = 4) and retinitis pigmentosa mice (P23H*/Gnat2^−^^/^^−^*, *n* = 4) at 1 month of age. One retina was used from each mouse. (**A**) A Uniform Manifold Approximation and Projection (UMAP) graph that was used for identifying the cell clusters. (**B**) A census plot of the cell clusters. (**C**) Cell counts and fractions in each cluster for both genotypes. A total of 5805 and 7266 and cells were captured and analyzed from *Gnat2^−^^/^^−^* and P23H*/Gnat2^−^^/^^−^* mouse groups, respectively. Acs, amacrine cells; CBC, cone bipolar cell; ECs, endothelial cells; GABA, gamma-aminobutyric acid; HCs, horizontal cells; RPE, retinal pigment epithelium; MGs, Müller glia cells; RBCs, rod bipolar cells.

The rod cluster covers two thirds of all captured cells (in total 9417 rods captured) where data were aggregated from both *Gnat2^−^^/^^−^* and P23H/*Gnat2^−^^/^^−^* mouse retina cells (Figs. 1A, 1B). This important cluster for our study is still sufficiently similar in size between genotypes (Fig. 1C), despite already ongoing rod degeneration in the P23H/*Gnat2^−^^/^^−^* mice.^20^ In order of size, the next largest clusters were Müller glia (MG) cell cluster 1 (577 total captured cells), RBCs (558 captured cells), and MG cluster 2 (411 captured cells). Although we focus our analysis and discussion solely on data from rod and RBC clusters in this article, the full dataset is made publicly available (see Supplementary Files S1, S2; GEO accession number GSE291972).

**Figure 2.**
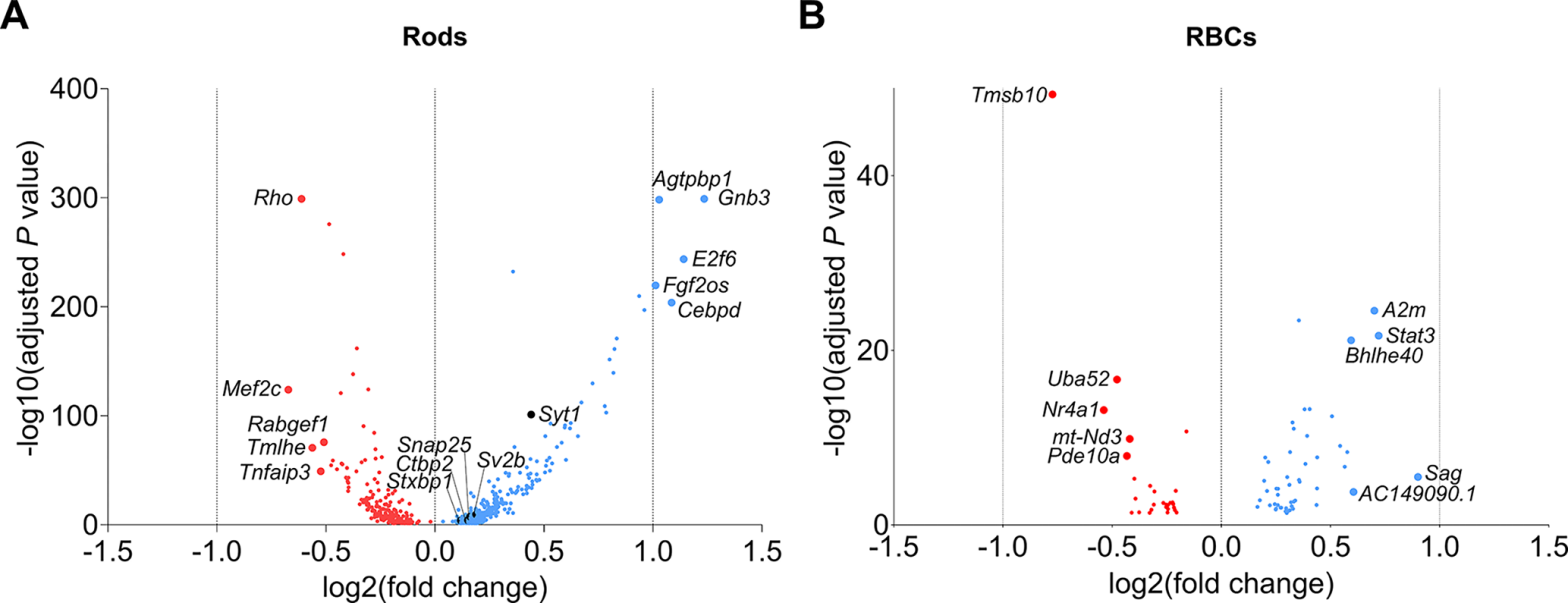
**Differentially expressed genes in rods and RBCs of P23H/Gnat2***^−^***^/^***^−^* **mice versus Gnat2***^−^***^/^***^−^* **mice**. Note, the y-axis is 8-fold larger in panel (**A**) compared to panel (**B**). The cutoff for DEGs was set at q < 0.05, with no fold-change criterion.

With a statistical cut off value of q < 0.05 (no fold-change criteria applied), we found 333 and 51 upregulated, and 209 and 28 downregulated, DEGs in rods (Fig. 2A; Supplementary File S3) and RBCs (Fig. 2B; Supplementary File S4), respectively. Importantly, upregulation is observed in several genes encoding synaptic soluble *N*-ethylmaleimide-sensitive factor attachment receptor (SNARE) complex proteins, such as *Snap25* (synaptosomal-associated protein 25), *Stxbp1* (syntaxin-binding protein 1, encodes STXBP1/MUNC18-1), and *Syt1* (synaptotagmin-1)*,* in the rods of P23H/*Gnat2^−^^/^^−^* mice (1.12-, 1.09-, and 1.36-fold upregulation, respectively; see Fig. 2A). The Kyoto Encyclopedia of Genes and Genomes (KEGG) pathway analysis detects an “upregulated glutamatergic synapse” pathway in the rod cluster but not in the RBC cluster (Supplementary Figs. S1, S2). The same pathway is prominently upregulated in the bulk RNA-seq of 1-month-old P23H retinas, as shown previously (see Fig. 3C in Ref. 20). In contrast, we did not find altered genes in RBCs, which would directly imply postsynaptic scaling (Fig. 2B). It is notable that the captured rod cell count (*n* = 9417) is 16.9-fold higher than the RBC count (*n* = 558; see Fig. 1C), which could bias the dataset toward more DEGs in the rod cells. Nevertheless, the collective scRNA-seq data indicate that presynaptic scaling of rods could more likely explain upscaled rod-RBC synaptic transmission^20^ than postsynaptic scaling at the level of RBCs in P23H/*Gnat2^−^^/^^−^* mice.

**Figure 3.**
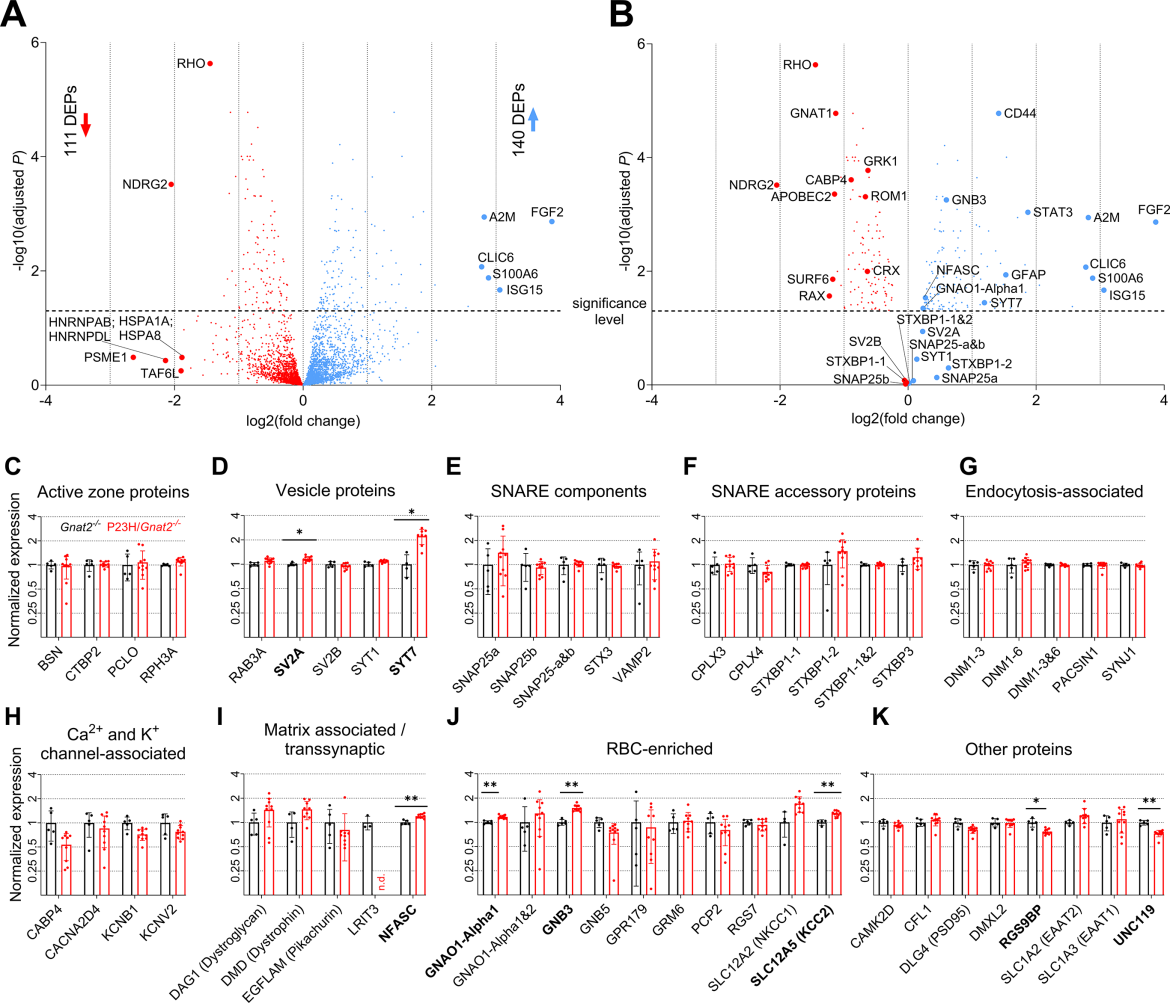
**Differentially expressed proteins in whole retinal extracts from P23H/Gnat2***^−^***^/^***^−^* **mice compared** **with** **Gnat2***^−^***^/^***^−^* **mice.** (**A**) Volcano plot containing all differentially expressed proteins (DEPs). Highlighted are the top five up- and downregulated proteins based on fold-change, as well as RHO as a relevant marker protein in retinas from P23H-RHO-mutant mice. (**B**) Replicated volcano plot where only selected, or significantly regulated, proteins are shown along with the top five proteins based on fold-change. The statistical analysis was performed with the limma package in Perseus. The cutoff for DEPs was set at q < 0.05 with no fold-change criterion. (**C-K**) Scatter plots of proteins of interest regardless of results from statistical analysis. Statistically significant DEPs are highlighted with * (q < 0.05) and ** (q < 0.01). The scatter plots include mean ± SD markers.

### Bulk Retina Proteome Analysis Fails to Detect Significant Regulation of SNAP25, STXBP1, or SYT1 in the Retinas of P23H/Gnat2*^−^*^/^*^−^* Mice

Bulk retinal omics analyses are inherently prone to false negatives in detecting cell-type- or layer-specific changes, due to masking or dilution effects. For example, a gene or protein modestly altered in a single cell class may be easily diluted in whole-tissue data, whereas opposing changes, such as upregulation in the outer plexiform layer (OPL) and simultaneous downregulation in the inner plexiform layer (IPL), could result in an apparent lack of net change.^36^ Given the current absence of high-throughput methods to assess spatially localized protein expression, we nonetheless sought to determine whether the same SNARE-complex components identified as upregulated in rods at the mRNA level (see Fig. 2A) are also altered in bulk retinal proteomics of whole P23H/*Gnat2^−^^/^^−^* retinas. Importantly, in both young P23H/*Gnat2^−^^/^^−^* and control retinas, the majority of protein signals are still expected to derive from the predominant cell population, that is, rod photoreceptors (see Figs. 1B, 1C).

With a statistical cut off value of q < 0.05 (no fold-change criteria applied), we found 111 downregulated and 140 upregulated proteins in the retinal extracts from P23H/*Gnat2^−^^/^^−^* mice compared with *Gnat2^−^^/^^−^* mice (Fig. 3A). The majority of the downregulated proteins are photoreceptor-specific, such as rhodopsin (RHO), guanine nucleotide-binding protein G(t) subunit alpha-1 (GNAT1), retinal-specific phospholipid-transporting ATPase (ABCA4), rod outer segment membrane protein 1 (ROM1), and G protein-coupled receptor kinase 1 (GRK1), as well as several cyclic nucleotide-gated channels (CNGs) and guanylate cyclase activators (GUCAs; (Fig. 3B; Supplementary Files S5, S6), as expected due to anatomic rod degeneration.^20^ Rod degeneration also triggers a robust retinal-cell stress response in the retinas of P23H/*Gnat2^−^^/^^−^* mice; that is, the list of most upregulated proteins is dominated by cell stress and inflammation markers, such as fibroblast growth factor 2 (FGF2), interferon-stimulated gene 15 (ISG15), alpha-2-macroglobulin (A2M), signal transducer and activator of transcription 3 (STAT3), glial fibrillary acidic protein (GFAP), and extracellular matrix receptor III (CD44), among others. However, some of the findings (Figs. 3C–K) may be important for adaptive plasticity. For example, SYT7, which has the highest gene expression in amacrine cells^42^ (Supplementary Fig. S3, Supplementary File S7) and is robustly labeled in the IPL of mouse retinas,^43^ is overexpressed by more than 2-fold in the retinas of P23H/*Gnat2^−^*^/^*^−^* mice (see Fig. 3D). Neurofascin (NFASC), a cell adhesion molecule that localizes in rod and cone bipolar cells and in horizontal-cell processes,^44^ is overexpressed 1.2-fold in the retinas of P23H/*Gnat2^−^*^/^*^−^* mice (see Fig. 3I). For guanine nucleotide-binding protein G(o) subunit alpha (GNAO1), which has the highest expression in RBCs,^45^ we detected 2 different isoforms and one of these, P18872-1, was overexpressed 1.17-fold in the retinas of P23H/*Gnat2^−^*^/^*^−^* mice (see Fig. 3J). Guanine nucleotide-binding protein subunit beta-3 (GNB3), which is mostly expressed in cones, cone bipolar cells, and RBCs,^46^^,^^47^ is overexpressed 1.50-fold (see Fig. 3J). Furthermore, significant retinal remodeling is triggered in the retinas of P30 P23H/*Gnat2^−^*^/^*^−^* mice, as corroborated by altered KEGG and Gene Ontology (GO) pathways: “regulation of actin cytoskeleton,” “glutamatergic synapse,” “axon guidance,” and “synaptic vesicle cycle,” among others (Supplementary Figs. S4, S5).

If the expression of several functionally linked proteins is congruent, that is, moves in the same direction (toward down- or upregulation), the trend could be biologically meaningful. Therefore, we organized the normalized raw LFQ levels of the selected proteins (rationale for selection is explained in Supplementary File S8) into scatter plots for easily accessible visual evaluation (see Figs. 3C–K). For the SNARE and SNARE accessory proteins SNAP25 and STXBP1, we detected three different sequences, consisting of two separate isoforms and their aggregate. In the retinas of P23H/*Gnat2^−^^/^^−^* mice compared with those of *Gnat2^−^^/^^−^* mice, the relative total expression of SNAP25a (P60879-2), SNAP25b (P60879-1), and SNAP25-a and b (P60879-1 and P60879-2) are, on average, 1.41-, 0.92-, and 1.03-fold, respectively, whereas the relative expression of STXBP1-1 (O08599-1), STXBP1-2 (O08599-2), and STXBP1-1 and 2 (O08599-1 and O08599-2) are, on average, 0.98-, 1.48-, and 1.00-fold, respectively (see Fig. 3B; Supplementary File S9). The vesicle protein SYT1 is expressed, on average, 9% higher in the retinas of P23H/*Gnat2^−^*^/^*^−^* mice compared with *Gnat2^−^*^/^*^−^* mice.

### Immunohistochemistry Suggests Increased SYT1 and SNAP25 Expression in the Surviving Rod Terminals of P23H/Gnat2*^−^*^/^*^−^* Mice

**Figure 4.**
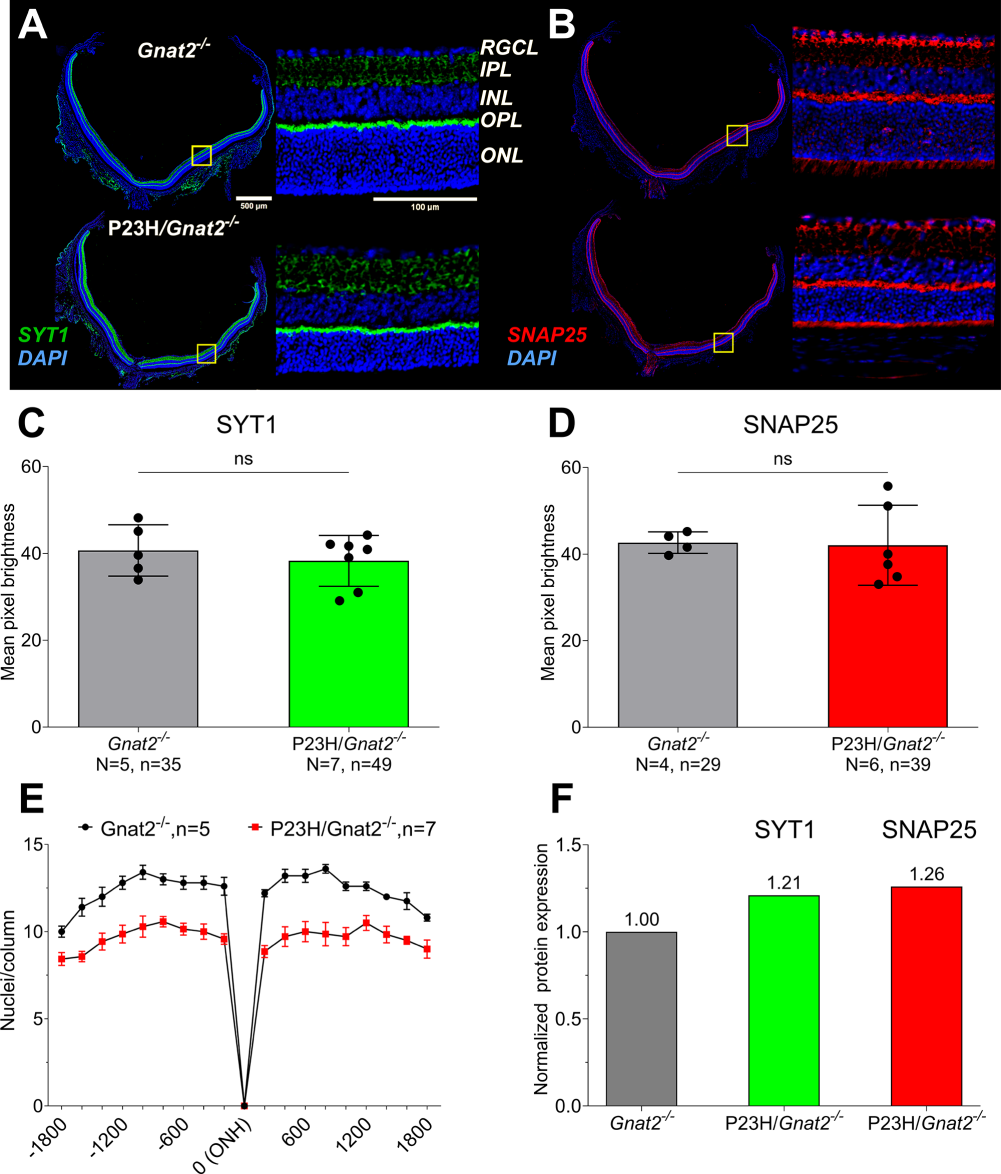
**SYT1 staining in whole retinas from** ***Gnat2**^**−**^*^**/**^*^−^* **control mice compared with P23H/*****Gnat2**^−^*^**/**^*^−^* **mice.** (**A**, **B**) Representative examples of SYT1 and SNAP25 staining (SYT1 green and SNAP25 red) merged with a nuclear stain (DAPI, blue) in retinas from 1-month-old *Gnat2^−^*^/^*^−^* and P23H/*Gnat2^−^*^/^*^−^* mice. (**C,** **D**) Mean pixel brightness in SYT1 and SNAP25 staining, respectively. (**E**) Photoreceptor nuclei count per ONL column at 18 points ranging from the optic nerve. (**F**) OPL SYT1 and SNAP25 signals normalized to average ratio of P23H/*Gnat2^−^*^/^*^−^* to *Gnat2^−^*^/^*^−^* ONL photoreceptor nuclei count. Data expressed as mean ± SD (**C,** **D**) and mean ± SEM (**E**).

The bulk retina proteomics failed to provide a definitive answer whether SYT1 and/or SNAP25 content is altered in the P23H/*Gnat2^−^*^/^*^−^* mouse retinas. Thus, we next sought to perform an OPL-specific protein expression analysis using immunohistochemistry (IHC), targeting this retinal region where rod–RBC synapses are located. Protein expression quantity can be roughly estimated from IHC staining intensity (i.e. optical density); however, this method is prone to high technical variability. Therefore, rather than relying on sampling from a few locations per retina, we analyzed the staining intensity through the entire length of the vertically oriented retina (Supplementary Figs. S6, S7), centered with respect to the naso-temporal orientation. Furthermore, at least two technical replicates (usually 6–7 replicates; Supplementary File S10) per retina were used, and the average intensity of these replicates was used in the statistical analysis. The mean pixel brightness of SYT1 and SNAP25 staining in the OPL of P23H/*Gnat2^−^*^/^*^−^* and *Gnat2^−^*^/^*^−^* mouse groups were practically the same (Figs. 4C, 4D; see Supplementary File S10), however, when the 22% outer nuclear layer (ONL) cell loss (mean value, Fig. 4E; see Supplementary Fig. S8) is accounted for, an average of 1.21- and 1.26-fold increase in P23H/*Gnat2^−^*^/^*^−^* mice per remaining rod cell is obtained for SYT1 and SNAP25 signal, respectively (Fig. 4F). We also intended to perform the same analysis for STXBP1, but due to the suboptimal quality of the staining (Supplementary Fig. S9), mean pixel brightness could not be reliably analyzed.

## Discussion

Synaptic scaling has been suggested as a possible mechanism explaining potentiation of the rod-RBC signal transmission during RP in the P23H/*Gnat2^−^*^/^*^−^* mouse model.^20^ Based on the single-cell gene expression data presented herein, we postulate that the remaining rod synaptic terminals may gain strength by upregulating the expression of genes encoding synaptic SNARE-complex and vesicle proteins, including *Snap25* and *Stxbp1,* and *Syt1* and *Sv2b* (synaptic vesicle glycoprotein 2B), respectively*.* Although we failed to detect significant upregulation of the SNAP25, STXBP1, or SYT1 proteins in whole retinal extracts using proteomics, the trend toward upregulation of these proteins (see Fig. 3), along with retained staining of SYT1 and SNAP25 in the OPL despite dying rods (see Fig. 4), as well as increased *Syt1* and *Snap25* mRNA in individual rods (see Fig. 2A), supports the hypothesis of presynaptic scaling in P23H/*Gnat2^−^^/^^−^* mouse retinas. Conversely, we did not observe clearly altered gene expression profiles in RBCs that would imply significant postsynaptic scaling (see Fig. 2B). However, in the previous study, genes such as *Grm6* and *Trpm1*, essential for ON bipolar cell depolarization, were upregulated in 1-month-old P23H retinas.^20^ This discrepancy could have occurred for several reasons. First, in the previous paper, bulk RNA-seq was used instead of scRNA-seq, and it is possible that the scRNA-seq is not sufficiently sensitive to detect *Grm6* or *Trpm1* upregulation in any of the depolarizing bipolar cell types if the total upregulation is spread among the various cell types. Second, the current scRNA-seq was performed using retinas from P23H*/Gnat2^−^^/^^−^* mice, whereas retinas from P23H single-mutant mice were used in the earlier study using bulk RNA-seq.

SYT1 is a vesicular, low-affinity Ca^2+^ sensor that is expressed in both conventional and ribbon synapses.^48^ It plays a crucial role in fast SNARE-mediated exocytosis in photoreceptors.^49^ SNAP25 is a synaptosomal protein that localizes to the presynaptic plasma membrane^50^ and mediates Ca^2+^-dependent exocytosis by facilitating SYT1 binding to SNARE complexes.^51^ It is necessary for the maturation and survival of photoreceptors.^52^ STXBP1 accelerates SNARE-dependent membrane fusion^53^ and is required for the proper localization of synaptosomal proteins, for example, SNAP25, within photoreceptor synaptic terminals.^54^ In addition to SYT1, another important vesicle component is SV2B, which acts as a positive modulator of rod-RBC neurotransmission and is the primary isoform in rod synaptic terminals.^55^ *Sv2b* gene expression is significantly upregulated in P23H/*Gnat2^−^*^/^*^−^* mouse rods (see Fig. 2A), while the protein expression remains nearly unchanged in bulk retina proteomics (see Fig. 3D) despite significant rod loss. Together with the upregulated SNARE complex components, increased expression of this synaptic vesicle component could enable enhanced capacity for glutamate release from surviving P23H/*Gnat2^−^*^/^*^−^* mouse rods, particularly when considering the interaction with SYT1.^56^ However, further research is required to functionally evaluate glutamate release properties. Notably, another synaptotagmin protein SYT7 is upregulated two-fold in P23H/*Gnat2^−^*^/^*^−^* mouse retinal extracts compared with controls (see Fig. 3D). Although SYT7 is not crucial for mouse rod-RBC synaptic function, it does play a modulatory role in the rod pathway, as global SYT7 knockout attenuates scotopic ERG b-waves and oscillatory potentials.^43^

Our proteomics data show upregulation of many matrix-associated/transsynaptic-complex proteins in P23H/*Gnat2^−^*^/^*^−^* versus *Gnat2^−^*^/^*^−^* mouse retinas, which further implies strengthening of rod-RBC synapses in P23H/*Gnat2^−^*^/^*^−^* mice. For example, RBC-enriched NFASC is overexpressed in P23H/*Gnat2^−^*^/^*^−^* mouse retinas (see Fig. 3I). Although the exact role of this protein in the rod pathway is not known, it is necessary for the expression of at least two crucial pre- and postsynaptic proteins at the rod-RBC synapse; namely, bassoon (BSN) and metabotropic glutamate receptor 6 (GRM6), respectively.^44^ The expression patterns of dystrophin (DMD) and dystroglycan 1 (DAG1) also suggest a meaningful modulation at the rod-RBC synapse, as these physiologically interacting presynaptic extracellular matrix (ECM) proteins regulate the synaptic connection between rods and RBCs,^57^^,^^58^ and are crucial for rod-RBC signal transfer.^57^^,^^59^^,^^60^ Compensatory synaptogenesis between rods and RBCs has not been supported previously,^20^ or by our current data. However, there exists a discrepancy between the 4% increase in GRM6 expression in the retinas of P23H/*Gnat2^−^*^/^*^−^* mice and the previously observed approximately 20% decrease in photoreceptor-RBC synapse count.^20^ This finding could be best explained by denser postsynaptic clustering of GRM6 in the remaining rod-RBC synapses.

Finally, although the current data supports presynaptic scaling, the potential influence of postsynaptic mechanisms or intrinsic properties of rods and RBCs should not be overlooked. For instance, the significantly upregulated proteins GNAO1-Alpha1 (P18872-1) and GNB3 (see Fig. 3J) could imply enhanced signaling in the transduction cascade of postsynaptic GRM6 receptors in RBCs. In addition, the RBC cascade is physically linked to the presynaptic-release machinery through the trans-synaptic-protein complex, and these interactions play a key role in maintaining rod-RBC synapses.^61^^,^^62^ Thus, a comprehensive view of homeostatic compensation can be achieved only by considering the reciprocal molecular cascade within the rod-RBC synapse, including the trans-synaptic-complex and its possible role in shaping the synaptic architecture and synaptic responses.^63^ More research on these plasticity mechanisms is needed to optimally utilize homeostatic plasticity in vision-saving therapies while simultaneously suppressing maladaptive retinal remodeling.^64^ Another important aspect to study in the future is the effect of aging, as plasticity typically declines with age. Indeed, reduced retinal plasticity has been observed in mature mouse retina,^65^ although, in another test paradigm, adult mouse retinas still exhibited considerable functional compensation.^66^

### Limitations of the Data

Although scRNA-seq enables the detection of DEGs related to synaptic scaling in both rods and RBCs, the disproportionately high number of captured rods compared with RBCs results in greater statistical power in the rod cluster, leading to the identification of more DEGs. A major limitation of global bulk proteomics is the poor coverage of membrane proteins, such as G protein-coupled receptors and ion channels, and the lack of spatial resolution in protein localization. Proteins embedded in membranes are known to be difficult to separate and identify, which makes them difficult to study.^67^ Further data on the expression and localization of these, and proteoforms in general, could provide valuable insights into presynaptic homeostatic plasticity and its underlying molecular mechanisms. For example, the calcium channel voltage-dependent L-type calcium channel subunit alpha-1F, Ca_V_1.4 (CACNA1F), which was not detected in our proteomics dataset but plays a crucial role in organization of the rod photoreceptor synapse,^68^ has also been shown to drive presynaptic plasticity at the cone-photoreceptor synapse.^24^ Moreover, changes in the expression or post-translational modification of, for example, GRM8 (metabotropic glutamate receptor 8) that is an inhibitory autoreceptor in rods,^69^^,^^70^ could be important in presynaptic scaling. In some cases, false negatives in layer-specific changes could be reduced by examining specific splice variants, such as PICCOLINO, the ribbon-specific variant of piccolo (PCLO ).^71^^,^^72^ In adddition, differentiating the expression of protein isoforms specifically in the OPL could increase knowledge on presynaptic homeostatic plasticity. For example, SNAP25 isoforms can regulate plasticity in a central synapse,^73^ and they have been suggested to play a role in photoreceptors as well.^74^ In addition, the gene-expression data (see Supplementary Fig. S3, Supplementary File S7) may not accurately correspond with the protein expression levels in specific cell types, for instance, due to low protein turnover, which decreases the requirement for translation. For example, *Bsn* is reportedly expressed only in 5.35% of rods (see Supplementary File S7^26^), although, in principle, the protein BSN is found in every rod photoreceptor terminal. Estimating the site of differential expression based on the scRNA-seq data is complicated by this discrepancy, thereby making it more difficult to predict the biological significance of DEPs identified in the global proteomics data. Finally, in our protein expression experiment based on IHC signal quantification, the linear relationship between fluorescence signal intensity and the actual abundance of target proteins was not independently validated, a common limitation inherent to immunohistochemistry.^75^

## Supplementary Material

Supplement 1

Supplement 2

Supplement 3

Supplement 4

Supplement 5

Supplement 6

Supplement 7

Supplement 8

Supplement 9

Supplement 10

Supplement 11
