# Supplementary material for: Presynaptic Changes in Mouse Rod Photoreceptors During Early Retinitis Pigmentosa
Source: Invest Ophthalmol Vis Sci. 2025 Dec 1;66(15):4. doi: 10.1167/iovs.66.15.4 (PMC12697692; doi:10.1167/iovs.66.15.4)
Supplement: Supplement 1 [file iovs-66-15-4_s001.docx]

# **SUPPLEMENTARY FIGURES**

**Presynaptic changes in mouse rod photoreceptors during early retinitis**

**pigmentosa**

Elias Roihuvuo^1^, Lee Sturgis^2^, Ahmed B. Montaser^1^, Marcin Tabaka^2,3^, Elliot H. Choi^4^, Deepa Mathew^5^, Anthi-Styliani Makiou^1^, Kristiina M. Huttunen^1^, Krzysztof Palczewski^4,6,7,8^, Frans Vinberg^5^, Henri Leinonen^1*^

^1^School of Pharmacy, Faculty of Health Sciences, University of Eastern Finland, P.O. Box 1627, FI-70211 Kuopio, Finland.

^2^International Centre for Translational Eye Research, Warsaw, Poland.

^3^Institute of Physical Chemistry, Polish Academy of Sciences, Warsaw, Poland.

^4^Department of Ophthalmology, Gavin Herbert Eye Institute, University of California, Irvine, CA, USA.

^5^John A. Moran Eye Center, University of Utah, Salt Lake City, UT, USA

^6^Department of Physiology and Biophysics, University of California, Irvine, CA, USA.

^7^Department of Chemistry, University of California, Irvine, CA, USA.

^8^Department of Molecular Biology and Biochemistry, University of California, Irvine, CA, USA.

**Corresponding Author**:

Henri Leinonen

School of Pharmacy, Faculty of Health Sciences

University of Eastern Finland

P.O. Box 1627, FI-70211, Kuopio, Finland

[henri.leinonen@uef.fi](mailto:henri.leinonen@uef.fi)

Contents

[1. scRNA-seq GO and KEGG pathway analyses 3](#_Toc201440272)

[2. Genes expression of retinal markers used in this study 4](#_Toc201440273)

[3. Proteomics GO and KEGG pathway analyses 5](#_Toc201440274)

[4. Immunohistochemistry from retinal cross-sections 6](#_Toc201440275)

### scRNA-seq KEGG and GO pathway analyses


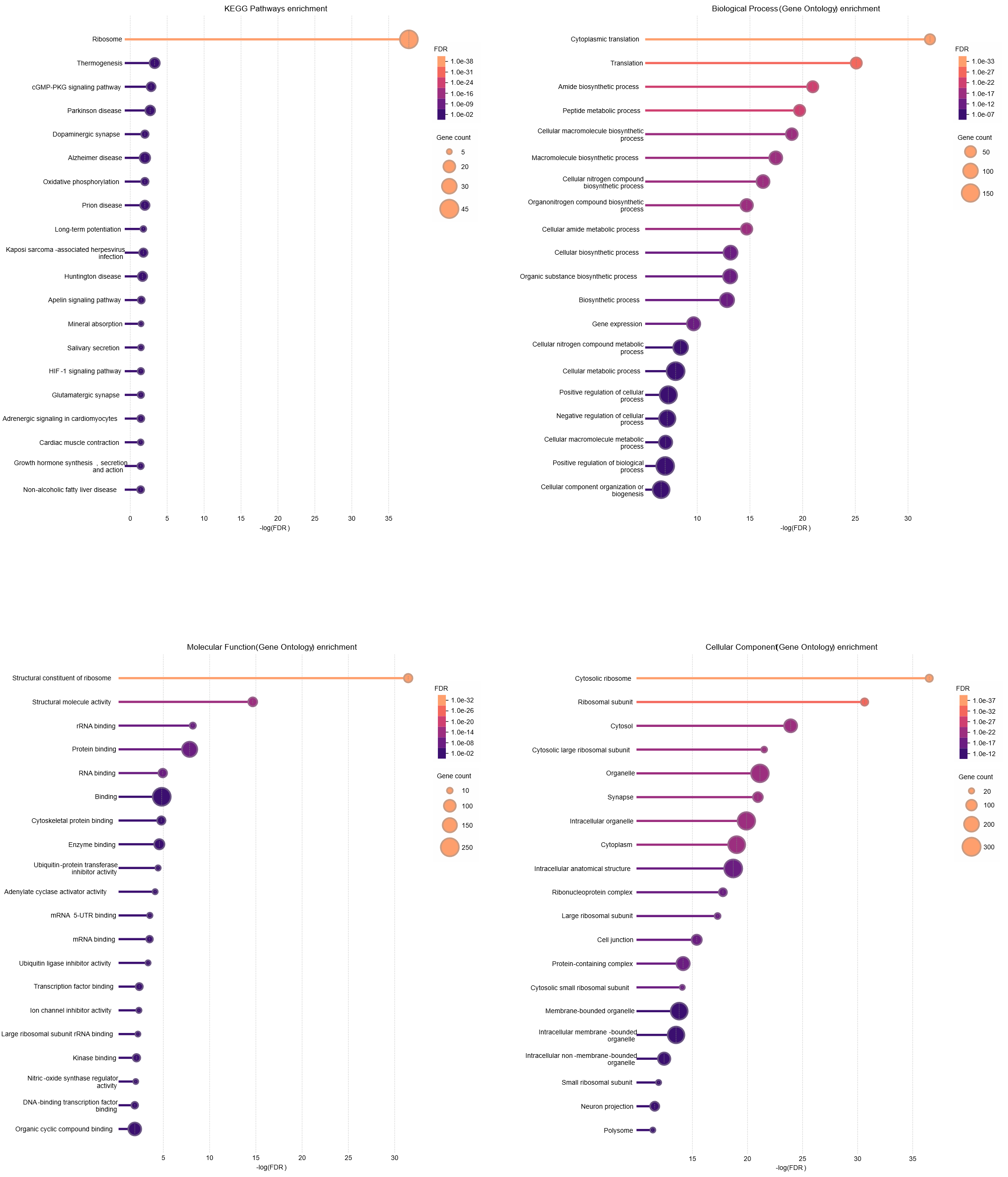


**Figure S1.** KEGG and GO pathway enrichment analyses based on upregulated DEGs in the rod cluster of P23H/*Gnat2⁻/⁻* mouse retinas relative to *Gnat2⁻/⁻* controls. The analysis is based on 333 query genes from string.db.


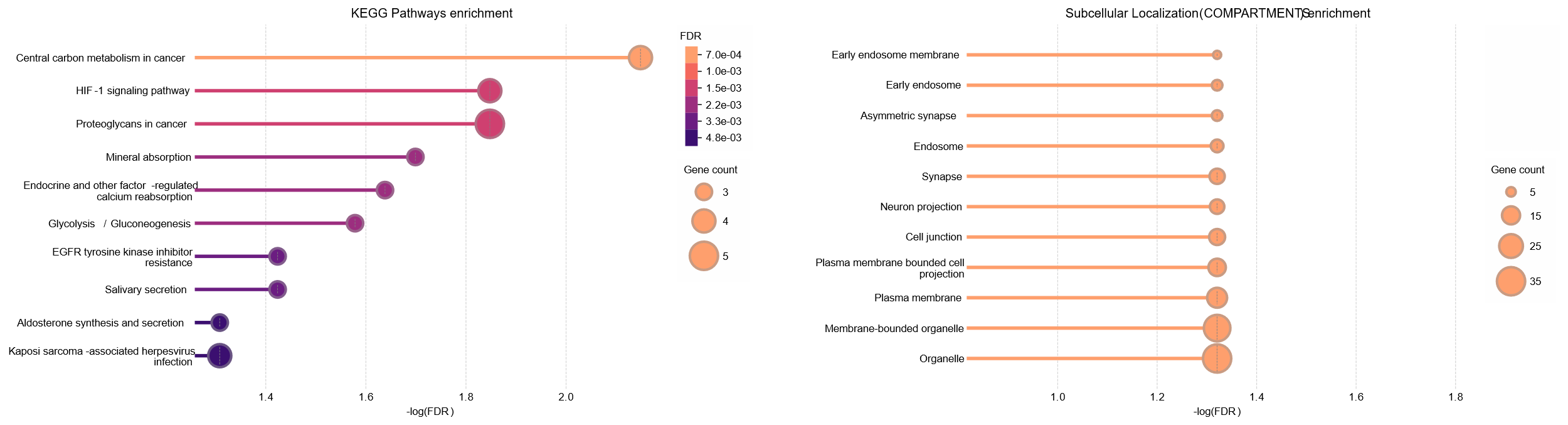


**Figure S2.** KEGG and GO pathway enrichment analyses based on upregulated DEGs in the rod cluster of P23H/Gnat2⁻/⁻ mouse retinas relative to Gnat2⁻/⁻ controls. The analysis is based on 51 query genes from string.db.

1. Genes expression of retinal markers used in this study
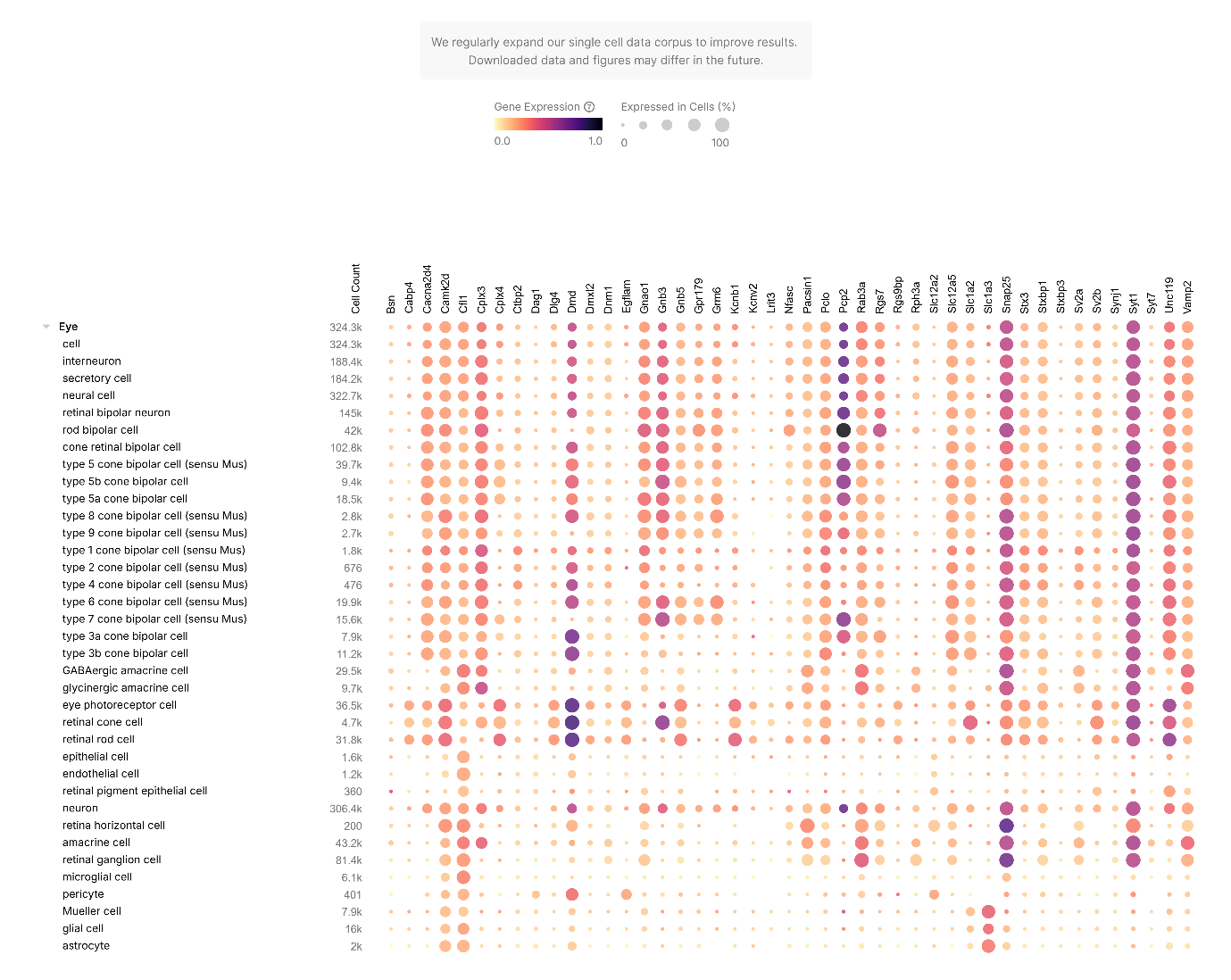


**Figure S3**. Mouse eye gene expression dot plot downloaded from CellxGene online tool: (<https://cellxgene.cziscience.com/gene-expression> doi: https://doi.org/10.1101/2023.10.30.563174, accessed: March 3, 2025)

### Proteomics KEGG and GO pathway analyses


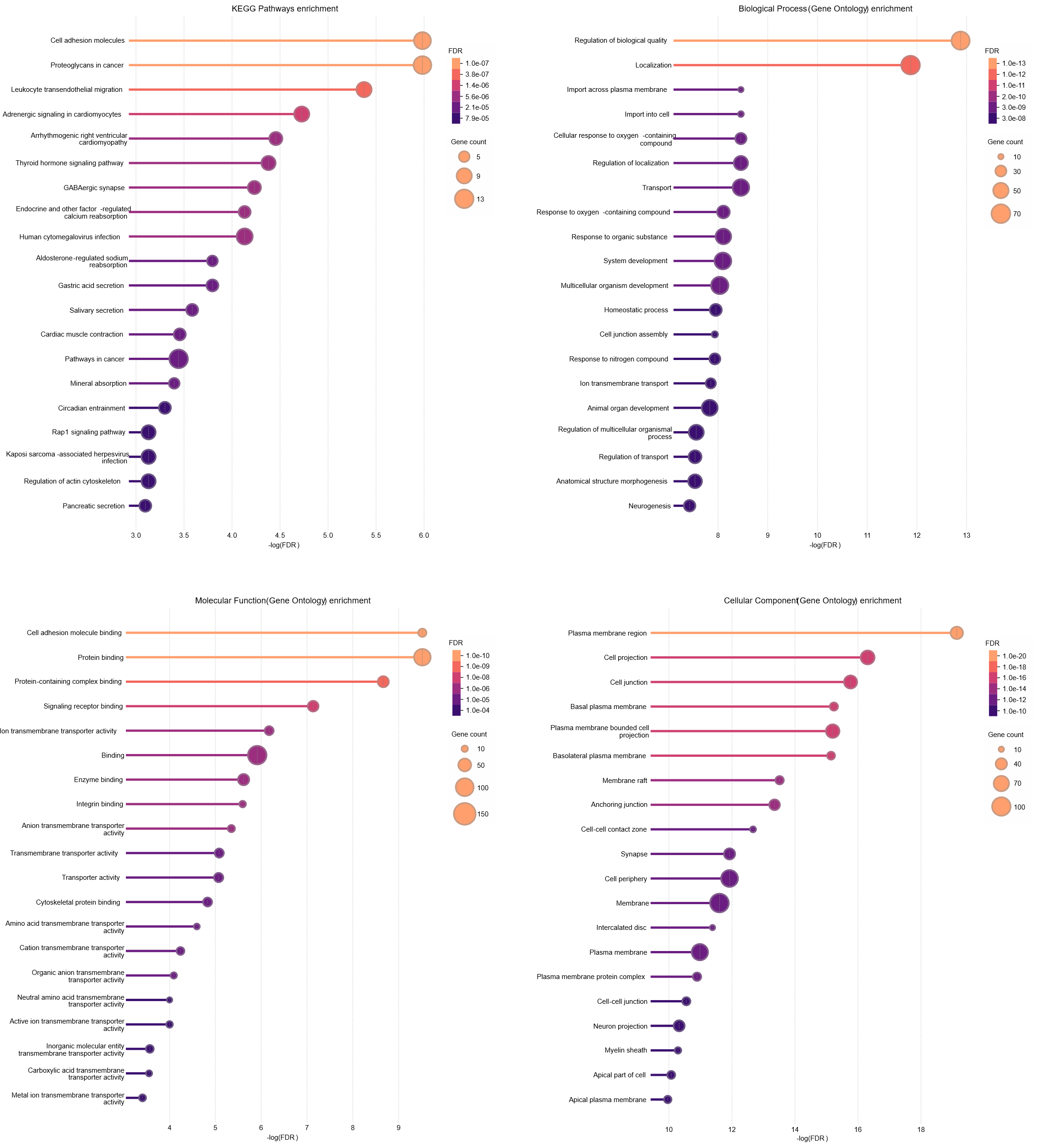


**Figure S4**. KEGG and GO pathway enrichment analyses based on proteins upregulated in P23H/Gnat2⁻/⁻ mouse retina homogenates relative to Gnat2⁻/⁻ controls. The analysis is based on 140 query proteins from string.db


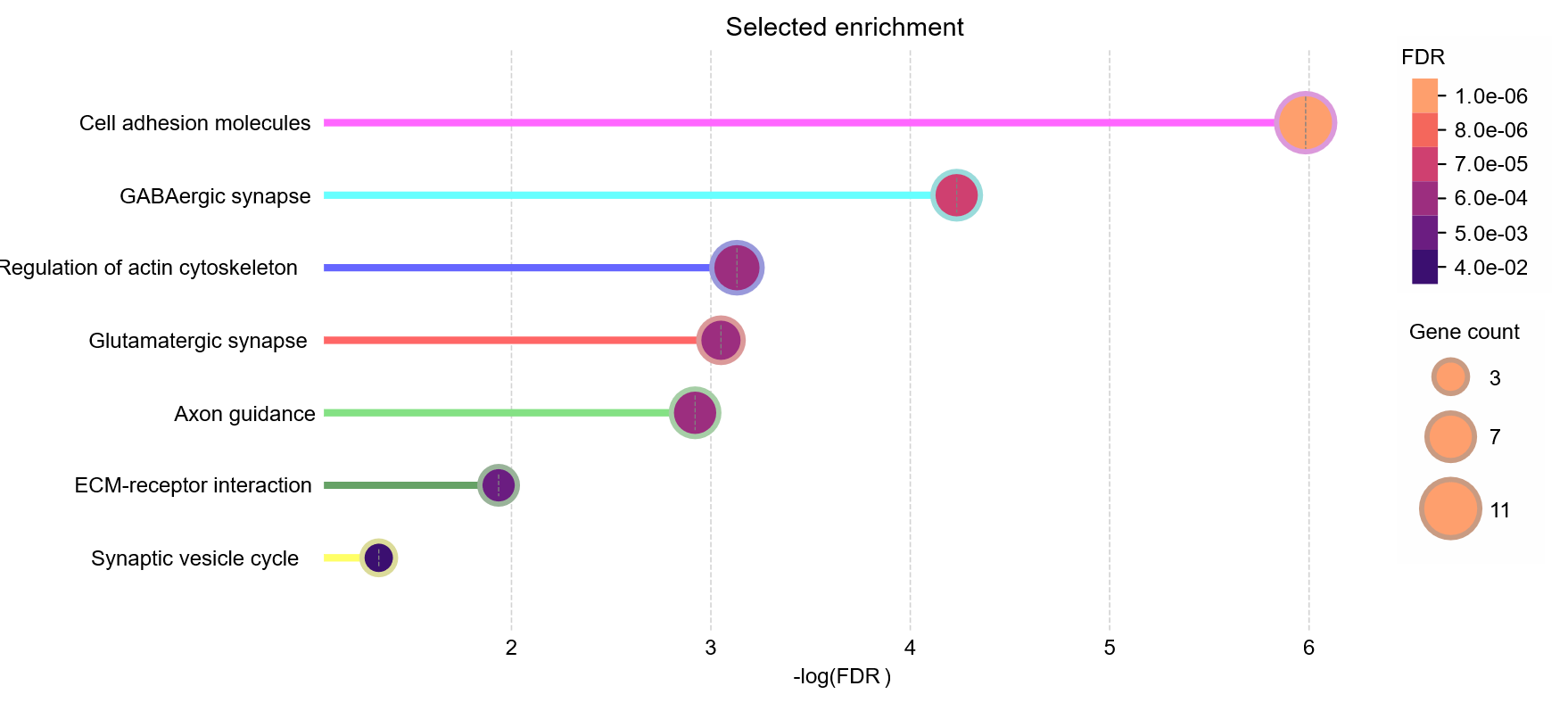


**Figure S5**. Selected GO pathway enrichment analyses based on proteins upregulated in P23H/Gnat2⁻/⁻ mouse retina homogenates relative to Gnat2⁻/⁻ controls. The analysis is based on 140 query proteins from string.db

### Immunohistochemistry from retinal cross-sections


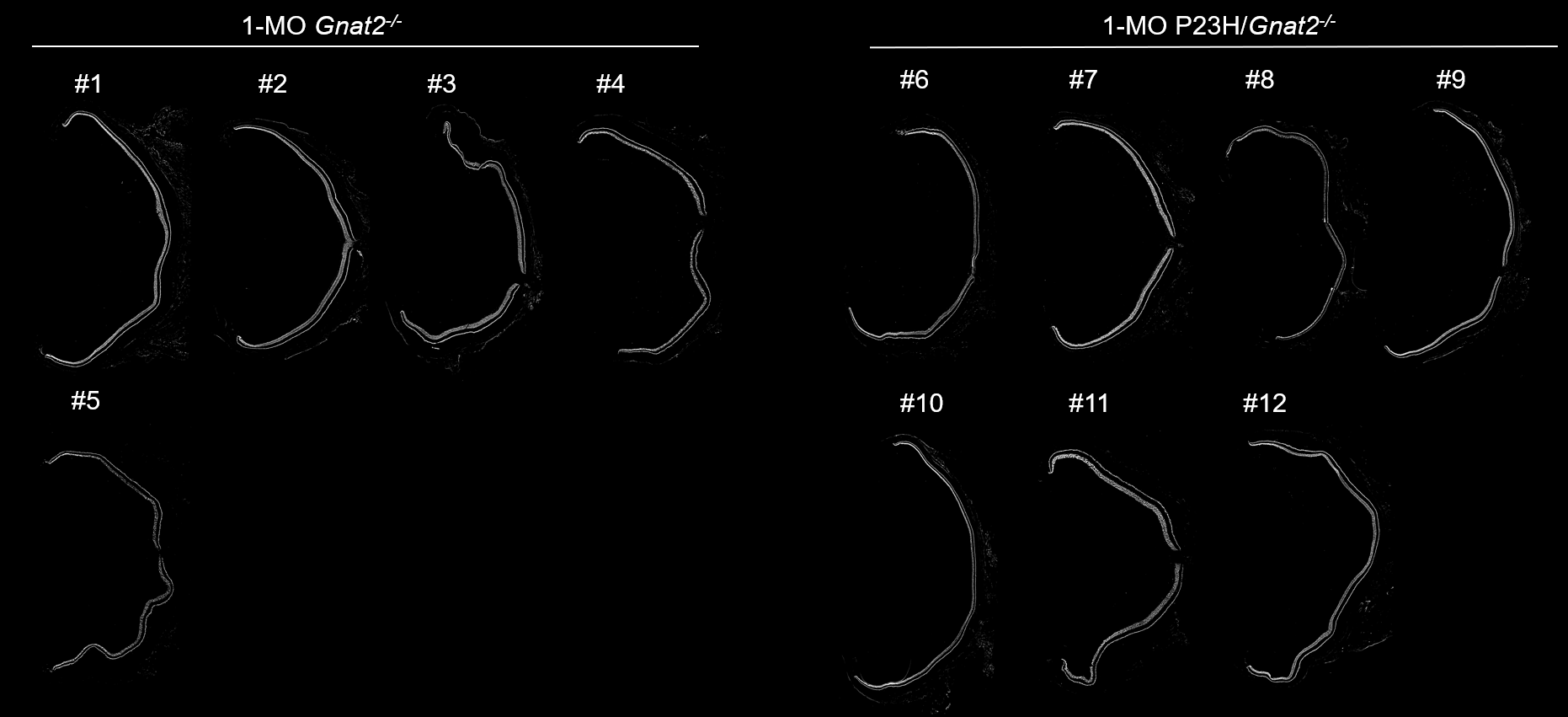


**Figure S6**. Representative retinal cross-sections from each biological replicate used for SYT1 expression analysis. SYT1 was labeled using an Alexa Fluor 488–conjugated secondary antibody. Sample numbers correspond to the unique identifiers of individual biological replicates.


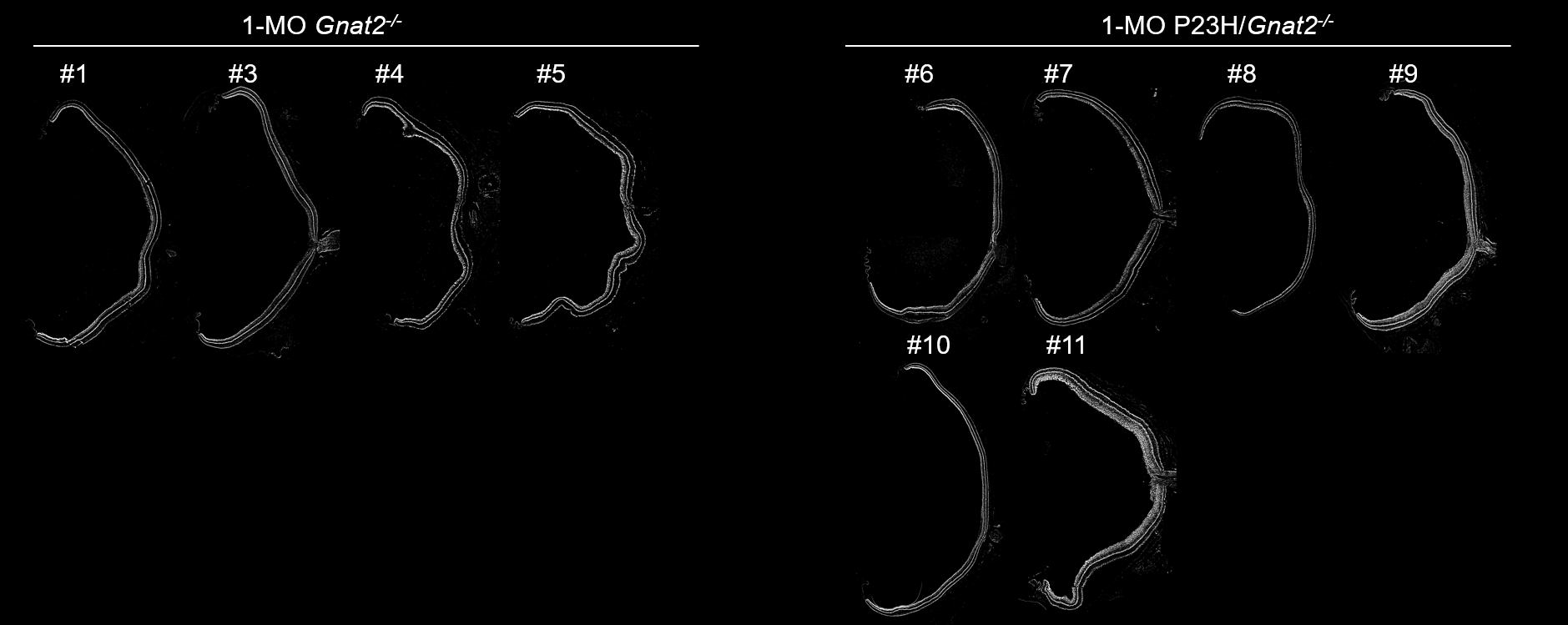


**Figure S7**. Representative retinal cross-sections from each biological replicate used for SNAP25 expression analysis. SNAP25 was labeled using an Alexa Fluor 647–conjugated secondary antibody. Sample numbers correspond to the unique identifiers of individual biological replicates.


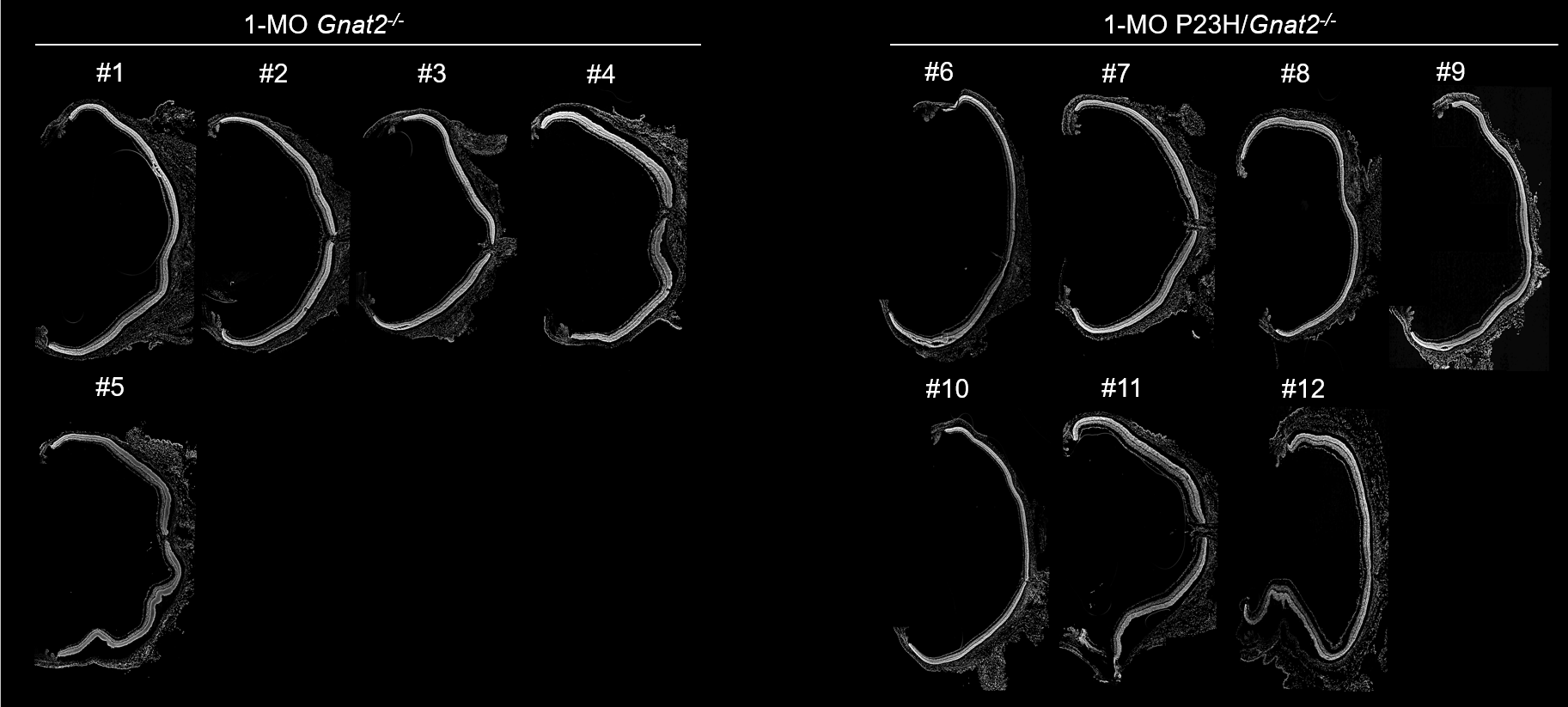


**Figure S8**. Representative images of retinal cross-sections from each biological replicate used for ONL thickness analysis. Nuclei were stained with DAPI. The sample number refers to the unique identifier of each biological replicate.


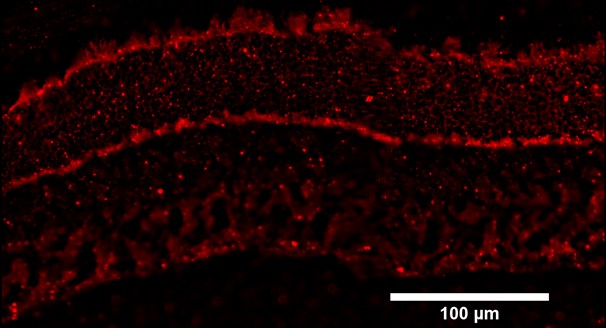


**Figure S9**. STXBP1/MUNC18-1 staining showing diffuse, granulose artifacts in mouse retina cross-section.

**Supplementary Datafile S1**. [scRNA-seq, DEGs for cluster annotation]

**Supplementary Datafile S2**. [scRNA-seq, Comparative gene expression in clusters 0-17]

**Supplementary Datafile S3**. [scRNA-seq_Cluster 0 (Rods)]

**Supplementary Datafile S4**. [scRNA-seq_Cluster 2 (RBCs)]

**Supplementary Datafile S5**. [Proteomics, limma results]

**Supplementary Datafile S6**. [Proteomics, DEPs]

**Supplementary Datafile S7**. [CELLxGENE_gene_expression]

**Supplementary Datafile S8**. [List of functions of selected proteins]

**Supplementary Datafile S9**. [Fold-change of relevant & categorized proteins]

**Supplementary Datafile S10**. [IHC, SYT1 and SNAP25 expression, ONL nuclei row analysis]
